# Effectiveness and safety of VISULAS^®^ green selective laser trabeculoplasty: a prospective, interventional multicenter clinical investigation

**DOI:** 10.1007/s10792-022-02617-7

**Published:** 2022-12-26

**Authors:** Karin R. Pillunat, Florian T. A. Kretz, Stefan Koinzer, Christoph Ehlken, Lutz E. Pillunat, Karsten Klabe

**Affiliations:** 1 Department of Ophthalmology, Medical Faculty Carl Gustav Carus, Technische Universität Dresden, Fetscherstrasse 74, 01307 Dresden, Germany; 2 Precise Vision Augenärzte, Augentagesklinik Rheine, Osnabrücker Str. 233-235, 48429 Rheine, Germany; 3 Augenarztpraxis Am Dreiecksplatz/Kiel, Holtenauer Straße 1, 24103 Kiel, Germany; 4 Department of Ophthalmology, Universitätsklinikum Schleswig-Holstein, Arnold-Heller-Str. 3, 24105 Kiel, Germany; 5 Internationale Innovative Ophthalmochirurgie GbR, Martin-Luther-Platz 22/26, 40212 Düsseldorf, Germany

**Keywords:** Primary open-angle glaucoma (POAG), Selective laser trabeculoplasty (SLT), VISULAS^®^ green laser, Intraocular pressure (IOP) reduction, Adjunctive IOP-lowering therapy, Adverse events

## Abstract

**Purpose:**

To evaluate the effectiveness and safety of Selective Laser Trabeculoplasty (SLT) with the SLT mode of the VISULAS^®^ green laser in patients with primary open-angle glaucoma (POAG).

**Methods:**

This prospective, interventional multicenter clinical investigation included patients with POAG who either needed a treatment escalation because the individual intraocular pressure (IOP) target was not met or treatment initiation and had an IOP ≥ 17 mmHg at baseline in the study eye. The study was conducted in five research centers across Germany. Approximately 100 laser applications were delivered to 360° of the trabecular meshwork. Glaucoma medications were not modified during the 3-month follow-up to allow evaluation of the sole effect of VISULAS^®^ green with SLT. Efficacy outcomes were postoperatively absolute and relative IOP changes at 1 and 3 months. Safety outcomes analyzed the rate of intra- and postoperative adverse events.

**Results:**

Thirty-four eyes of 34 POAG patients were included. The overall mean number of preoperative glaucoma medications was 2.2 ± 1.4 in 29 treated eyes, 5 eyes were treatment naïve. Mean baseline IOP (mmHg) was 21.0 ± 2.69 and was reduced by − 3.53 ± 3.34 [95% CI − 4.61; − 2.45] and − 3.59 ± 3.41 [95% CI − 4.64; − 2.53] at the 1- and 3-month follow-up, respectively (*p* < 0.0001), with 48.5% of cases achieving a ≥ 20% IOP reduction at 3 months [95% CI = 30.8%; 66.5%]. The mean relative IOP reduction was − 16.4% and − 16.3% at 1 and 3 months, respectively (*p* < 0.0001). Potentially device- or procedure-related adverse events were mild to moderate and included 3 postoperative IOP-spikes and 6 reports regarding eye pain and discomfort. All were resolved without sequelae.

**Conclusions:**

SLT performed with the VISULAS^®^ green laser achieved clinically significant additional IOP reductions in medically treated as well as in treatment naïve eyes with POAG and there were no relevant safety issues. The results are comparable to other reported SLT studies.

## Introduction

The reduction in intraocular pressure (IOP) is currently still the only evidence-based therapy to treat patients with any form of glaucoma [1–3]. Maintaining visual function is the main treatment goal of this sight-threatening optic neuropathy that is caused by the damage and apoptosis of retinal ganglion cells, possibly leading to severe visual field defects. After two decades of clinical experience, selective laser trabeculoplasty (SLT) has gained in importance as a well-tolerated method to lower IOP in patients with primary open-angle glaucoma (POAG) and has recently been described as a "new star in glaucoma treatment" [4]. Its current appreciation is in part based on the technique's ability to reduce the dependency on topically administered anti-glaucomatous drugs—usually the first step in glaucoma management but hampered by high rates of non-adherence—or even become an alternative to medical glaucoma therapy.

SLT is usually performed with a frequency-doubled q-switched 532 nm Nd:YAG laser which selectively targets the pigmented trabecular meshwork while not affecting its other, non-pigmented structures or causing any permanent damage to the tissue [5]. The exact mechanisms of IOP reduction by SLT and the way it increases trabecular outflow are still not understood completely.

The effectiveness and safety of SLT has been demonstrated as primary [6–8] as well as adjunctive therapy [9, 10]. Current interest in this treatment option has in particular been sparked by the LiGHT trial (Laser in Glaucoma and Ocular Hypertension) [7, 8], which compared the efficacy of SLT versus eye drops as first-line treatment. At 36 months, 74.2% [95% CI = 69.3; 78.6] of patients in the SLT group required no drops to maintain intraocular pressure at target, which provided an argument for SLT as a first-line treatment in eyes with POAG and Ocular Hypertension [7]. At 6 years 69.8% of eyes in the SLT group required no medical or surgical treatment to remain at or below target IOP with less disease progression (*p* = 0.006), less need for incisional glaucoma surgery (*p* < 0.001) and cataract surgery (*p* = 0.03) [8].

The objective of the current study was to evaluate the effectiveness and safety of SLT with the VISULAS^®^ green laser in patients with POAG who either needed a treatment escalation because the individual target pressure was not met or treatment initiation and who had an IOP ≥ 17 mmHg. The VISULAS^®^ green laser is an integrated retina and glaucoma laser which can operate in a selective mode to perform SLT. It acts by selective photothermolysis and has a homogenous laser energy distribution. The applied laser energy is titrated according to the degree of angle pigmentation. To our knowledge, this is the first study to evaluate the efficacy and safety of SLT using the VISULAS^®^ green laser.

## Methods

This prospective, interventional multicenter clinical investigation was carried out in 5 research centers across Germany: Department of Ophthalmology, Universitätsklinikum Carl Gustav Carus (Dresden), Department of Ophthalmology, Universitätsklinikum Schleswig–Holstein (Kiel), Augentagesklinik Rheine (Rheine), Augenarztpraxis am Dreiecksplatz (Kiel) and Internationale Innovative Ophthalmochirurgie GbR (Düsseldorf).

The study was approved by the responsible Ethic Commission at the TU Dresden (MPG ff-EK-24012020) and the involved local Ethic Commissions at the participating study sites as well as by the German Competent Authority BfArM (EUDAMED: CIV-19–12-031,046). The study is registered at clinicaltrials.gov under NCT04519814. Written informed consent was obtained from all study participants.

### Eligibility criteria and study group

Thirty-four eyes of 34 White/European glaucoma surgery-naïve patients with POAG, aged ≥ 40 years, who either needed a treatment escalation (29 eyes, 85%) because the individual target IOP was not met or treatment initiation (5 eyes, 15%) and had an IOP ≥ 17 mmHg at baseline in the study eye, were included. Exclusion criteria were conditions that prevented contact lens stability or laser delivery, planned surgery within 3 months, any previous intraocular surgery except uncomplicated cataract surgery longer than 3 months prior to study entry, any previous laser trabeculoplasty, uveitis, any condition with the risk of developing neovascularization of the retina or iris, patients with psychiatric disorders or dementia, pregnancy and lactation. POAG was defined as having an optic disk with characteristic glaucomatous cupping and focal or diffuse thinning of the neuroretinal rim, and/or corresponding visual field defects with no other ocular or systemic diseases that might cause these defects, and an open anterior chamber angle with Shaffer grade 3 and/or 4 on gonioscopy. High pressure glaucoma (HPG) patients with a history of untreated IOPs higher than 21 mmHg, and normal pressure glaucoma (NPG) patients with a history of untreated IOPs equal or less than 21 mmHg, were included.

Target IOP was individually set as the upper limit of the IOP estimated to slow progression in such a way that vision-related quality of life be maintained for the life expectancy of the patient [11].

### Data collection and parameters

Baseline recordings included gender, age, non-ocular and ocular medical history such as number and classes of IOP-lowering medications, as well as previous surgeries. A thorough ophthalmic examination included refraction, best spectacle-corrected distance visual acuity (BCDVA), slit-lamp biomicroscopy of the anterior segment, undilated IOP measurements taken with Goldmann applanation tonometry (GAT), gonioscopy including scoring of pigmentation and dilated fundus examination with a 90-diopter lens. Automated perimetry was taken with the Swedish interactive threshold algorithm standard 30–2 program (Carl Zeiss Meditec Inc., Dublin, CA, USA).

Measurements of IOP were taken within a narrow time frame of the same 2 h of the day for each patient at each study visit in a sitting position using a Goldmann tonometer. Three consecutive measurements were performed in a masked, 2-person method and the median was taken.

For patients with only one eye eligible (i.e., meeting all the inclusion and exclusion criteria), the eligible eye was the study eye. For patients with both eyes eligible, the eye with higher IOP was the study eye. If both eyes had the same IOP, the right eye was selected as the study eye if the patient number was even; the left eye was selected as the study eye if the patient number was odd.

### Study intervention

VISULAS^®^ green (Carl Zeiss Meditec AG, Jena, Germany) is an integrated retina and glaucoma laser workplace operating with a diode-pumped frequency-doubled Nd:YVO_4_ laser at 532 nm wavelength. Depending on the selected treatment mode, VISULAS^®^ green can for instance be operated in photocoagulation mode for the treatment of different retinal pathologies or alternatively in selective mode (SLT) for the treatment of glaucoma.

In this study, treatment was performed using the new SLT mode of the VISULAS^®^ green laser, which applies laser pulses as a fixed multi-spot pattern consisting of 52 adjacent single pulses of squared spots sized 50 µm each forming an application of 400 μm in diameter, similar to conventional SLT lasers. The energy is set in µJ due to the small diameter of each single spot. The applied fluence or energy per area, which is the treatment relevant parameter [12], is the same as in conventional SLT lasers, however. The settings in µJ of the VISULAS^®^ green with SLT can thus be translated into mJ of conventional SLT lasers. The initial energy level was set according to the grade of angle pigmentation:

30–50 µJ pigmentation none to just visible (Scheie Grading: None).

20–30 µJ pigmentation mild to moderate (Scheie I + II).

10–20 µJ pigmentation marked to intense (Scheie III + IV).

The laser acts on the trabecular meshwork by selective photothermolysis. Approximately one hundred non-overlapping 400 μm applications were delivered in a single session to 360° of the trabecular meshwork, which was visualized with the Latina SLT goniolens (Ocular Instr., Bellevue, USA). The energy could be adjusted throughout the treatment based on patient pigmentation; microbubbles were not seen in any eye of any pigmentation grade.

All eyes undergoing SLT peri-operatively received either topical proxymetacaine hydrochloride (Proparacaine POS 0.5% AT, Ursapharm, Germany) or topical oxybuprocain hydrochlorid (Conjuncain EDO^®^, Bausch + Lomb, Dr. Mann Pharma, Germany). Following laser treatment, patients were given 2% sodium hyaluronate gel (Hylogel, Ursapharm, Germany) or similar to alleviate the discomfort (burning, scratching, foreign body sensation) possibly caused by the contact lens. No anti-inflammatory substances were applied. The number of glaucoma medications and substances was not changed or discontinued during follow-up to allow evaluation of the sole effect of VISULAS^®^ green with SLT.

Follow-ups took place one hour, one day, one month and 3 months postoperatively. The different parameters and examinations taken at these study visits are depicted in Table 1.

**Table 1:** Study visits and clinical examinations

Visits	Preop	Operative visit	1 h	1 Day	1 Month	3 Month
Screening	x					
Informed consent	x					
Demographics (Age, Gender)	x					
Ocular and non-ocular medical history (incl. glaucoma duration and treatment)	x					
Intraocular pressure (undilated)/mmHg	x		x	x	x	x
Visual field	x					x
BCDVA/log MAR	x				x	x
Pachymetry	x					
Gonioscopy incl. scoring of pigmentation	x					
Slit-lamp examination	x			x	x	x
Dilated fundus examination	x					
Surgical parameters (laser settings)		x				
Intraoperative events		x				
(Serious) adverse device effects		x	x	x	x	x
Ocular medications	x	x	x	x	x	x
Concomitant systemic medication	x		x	x	x	x

### Endpoints

The primary efficacy endpoint of the study was the mean change in absolute intraocular pressure (in mmHg) of the study cohort from baseline to month 1. Secondary efficacy endpoints were the mean change in absolute intraocular pressure (in mmHg) of the study cohort from baseline to month 3 and the mean change in relative intraocular pressure (in %) from baseline to month 1 and month 3. Additional exploratory efficacy endpoints were the rate of patients achieving 0 to < 10%, 10% to < 20%, and ≥ 20% IOP reduction from baseline at 1 and 3 months, respectively. Furthermore, the development of visual acuity and visual fields. Safety measures included postoperative IOP-spikes, device- or procedure-related adverse events, and severe adverse events over the entire course of the study.

### Statistical analysis

An independent statistician performed statistical analyses. Sample size estimation was performed with PASS 15 (PASS 15 Power Analysis and Sample Size Software (2017). NCSS, LLC. Kaysville, Utah, USA, ncss.com/software/pass). It was based on the primary effectiveness endpoint, i.e., the mean absolute change in IOP from baseline to month 1. According to Pillunat et al. [10], a mean absolute change from baseline of − 1.3 mmHg (SD = 1.8 mmHg) was found. To allow for a slightly less severely ill population, the mean absolute change from baseline was assumed to be − 1.1 mmHg. Based on this assumption, a sample size of 31 patients (i.e., study eyes) achieves 90% power to detect a mean absolute change from baseline of − 1.1 mmHg to month 1 with a two-sided level of significance of 5%.

Metric demographics, baseline characteristics and laser parameters were summarized using mean, standard deviation, minimum and maximum. Categorical parameters were summarized using N and the respective percentage. Exact Clopper-Pearson [13] 95% confidence intervals were computed for the proportion of patients with given ranges of IOP reduction from baseline at 1 and 3 months. The least-square mean was used to show the change in IOP from baseline at 1 and 3 months. Changes from baseline were analyzed using an ANCOVA model with baseline as cofactor. Analyses were performed using SAS 9.4 (SAS Institute, Cary, NC, USA). Plots were generated using R [14] version 3.3.3. A *p* value lower than 0.05 was considered as statistically significant.

## Results

Thirty-four eyes of 34 patients with POAG who had an IOP ≥ 17 mmHg (range 17.0–27.0) with or without IOP-lowering medication were included: five were treatment naïve eyes, and 29 were pre-treated eyes with on average 2.2 ± 1.4 IOP-lowering substances. Mean visual field parameters at baseline were: mean deviation (MD) − 3.8 ± 3.59 dB (range 0.8 to − 13.7; only one eye had a MD lower than − 12 dB) and pattern standard deviation (PSD) 4.4 ± 3.34 dB (range 1.1–11.9). Demographics, baseline characteristics, glaucoma preoperative assessments and laser parameters are summarized in Table 2.

**Table 2:** Demographic data, baseline characteristics and laser parameters of the study cohort

N. of eyes/patients	34/34
Age (years)	Mean ± SD: 64.7 ± 9.5
Eyes (right/left): N (%)	19 (55.9%)/15 (44.1%)
Gender (male/female): N (%)	20 (58.8%)/14 (41.2%)
Preoperative IOP (mmHg)	Mean ± SD: 21.0 ± 2.69
BCDVA (logmar)	Mean ± SD: 0.1 ± 0.17
C/D Ratio	Mean ± SD: 0.6 ± 0.18
MD (dB)	Mean ± SD: − 3.8 ± 3.59
PSD (dB)	Mean ± SD: 4.4 ± 3.34
Corneal pachymetry (µm)	Mean ± SD: 555 ± 34
Preoperative glaucoma medications	Mean ± SD: 2.2 ± 1.4
Medications: *N* (%)	PGA: 25 (73.5%)
	β-Bl: 19 (55.9%)
	α-Ag: 13 (38.2%)
	CAI: 17 (50%)
	Pilocarpin: 3 (8.8%)
Gonioscopy: N (%)	
Shaffer 3 (20–35°)	22 (64.7%)
Shaffer 4 (35–45°)	12 (35.3%)
Pigmentation: N (%)	
None	1 (2.9%)
I–just visible	5 (14.7%)
II–mild	16 (47.1%)
III–marked	12 (35.3%)
IV–intense	0
Pseudophacic: *N* (%)	6 (17.6%)
N. of laser spots	Mean ± SD: 100.7 ± 16
Average laser energy (µJ)	Mean ± SD: 32.7 ± 13.0
Area of treatment (°)	Mean ± SD: 357.4 ± 15.4

*N*—number, *IOP*—intraocular pressure, *BCDVA*—best-corrected distance visual acuity, *C*/*D*—*ratio* cup to disk ratio, *MD*—mean deviation, *PSD*—pattern standard deviation, *PGA*—prostaglandin analogs, β-*Bl*—beta-blockers, α-*Ag*—alpha-agonists, *CAI*—carbonic anhydrase inhibitors, *SD*—standard deviation

### Efficacy analysis

**Fig. 1:**
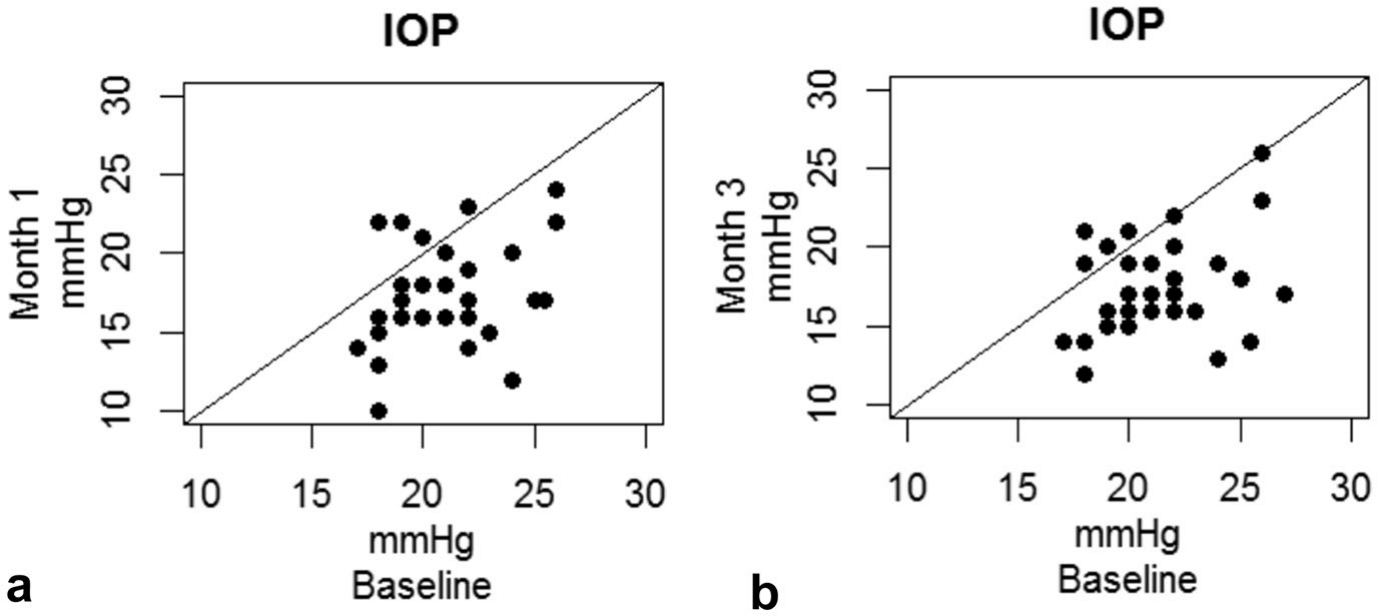
Scatterplots of absolute IOP values of month 1 **a** and month 3 **b** versus baseline. A higher baseline IOP is associated with a better IOP reduction

**Fig. 2:**
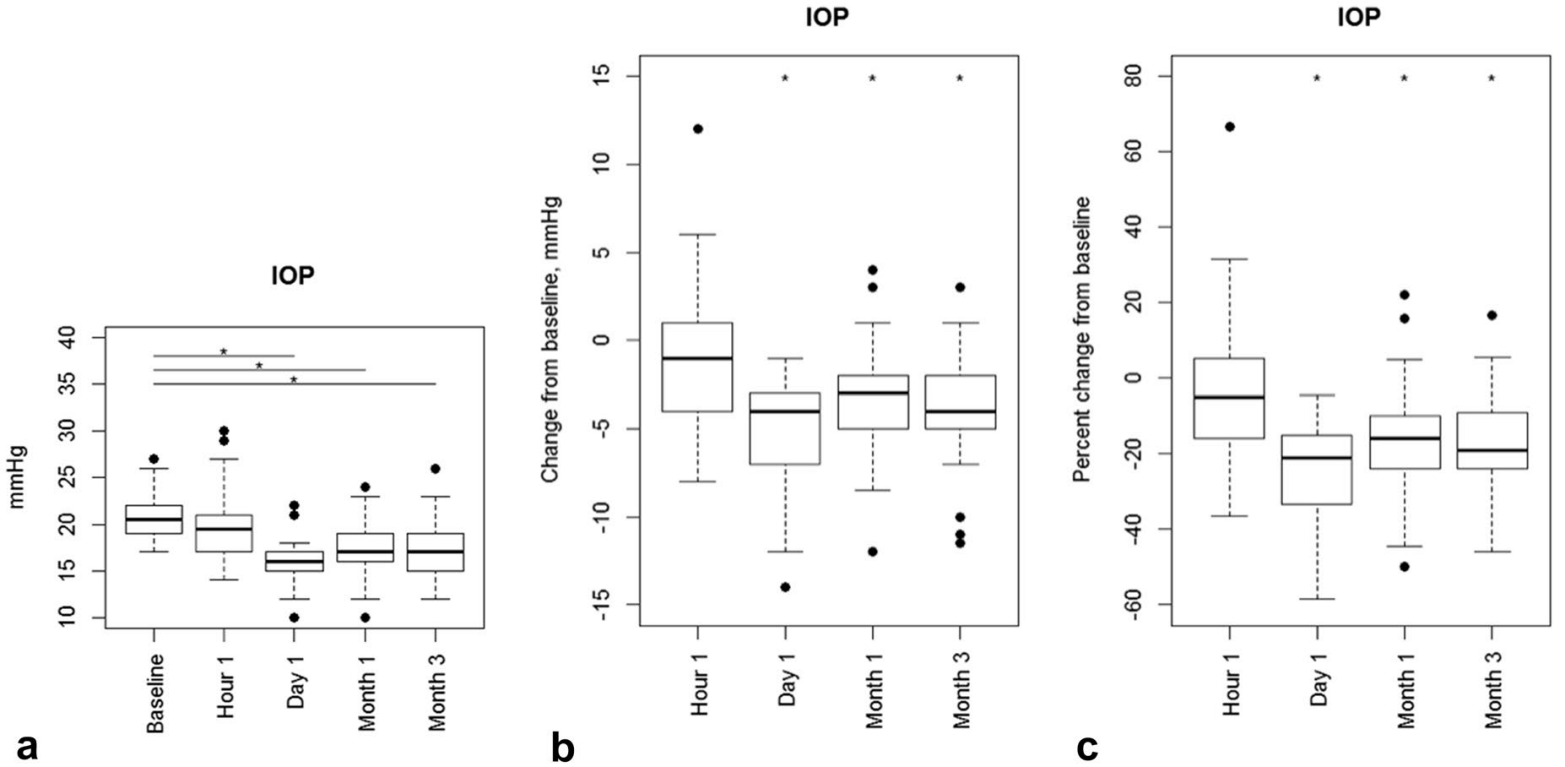
Boxplots showing the time course of absolute IOP values (left), the changes from baseline in mmHg (middle) and the percent changes from baseline (right). *Statistically significantly different

One month after treatment, IOP was statistically significantly reduced from a mean baseline IOP of 21.0 ± 2.69 mmHg (*n* = 34) to 17.3 ± 3.18 mmHg (*n* = 33; Table 3, Figs. 1a, and 2a, b) with least-square mean change of − 3.53 ± 3.34 mmHg (95% Confidence Interval, CI, [− 4.61; − 2.45], *p* < 0.0001). This IOP reduction remained stable throughout the 3-month follow-up with a least-square mean change of − 3.59 ± 3.41 mmHg (95% CI [− 4.64; − 2.53] *p* < 0.0001) to 17.5 ± 3.07 mmHg (*n* = 33; Table 3, Figs. 1b and 2a, b). The mean relative change was − 16.4% (95% CI [− 21.6; − 11.1] *p* < 0.0001) after 1 month and − 16.3% (95% CI [− 21.2; − 11.4], *p* < 0.0001) after 3 months (Table 3, Fig. 2c). Table 4 shows the proportions of participants achieving a ≥ 20%, between 10–20% and 0–10% as well as no IOP reduction at 1 and 3 months. After 3 months 48.5% (95% CI [30.8%; 66.5%]) of the patients reached a more than 20% IOP reduction from baseline.

**Table 3:** (Left) mean IOP at baseline and different follow-up time points. Means and standard deviation. (Right) mean absolute and relative change in IOP compared to baseline 1 and 3 months after treatment. Means and Clopper-Pearson 95% confidence intervals

Time point	Mean IOP [mmHg] (SD)	LS-Mean change [mmHg] (95% CI)	*p* value	LS-Mean change [%] (95% CI)	*p* value
Preop	21.0 (± 2.69)				
1 h	20.0 (± 3.77)				
Day 1	16.0 (± 2.45)				
Month 1	17.3 (± 3.18)	− 3.53 (− 4.61; − 2.45)	< 0.0001	− 16.4 (− 21.6; − 11.1)	< 0.0001
Month 3	17.5 (± 3.07)	− 3.59 (− 4.64; − 2.53)	< 0.0001	− 16.3 (− 21.2; − 11.4)	< 0.0001

*LS*-mean change = least-square mean change from baseline with baseline as cofactor, *CI*—confidence interval, *SD*—standard deviation

**Table 4:** Proportion of patients with ≥ 20%, 10—20%, 0—10%, and no IOP reduction from baseline at 1 and 3 months

Time point	IOP reduction	N	Proportion [Clopper–Pearson 95% CI]
month 1 (*N* = 33)	No	4	12.1% [3.4%; 28.2%]
	0–10%	3	9.1% [1.9%; 24.3%]
	10–20%	14	42.4% [25.5%; 60.8%]
	≥ 20%	12	36.4% [20.4%; 54.9%]
month 3 (*N* = 33)	No	7	21.2% [9.0%; 38.9%]
	0–10%	4	12.1% [3.4%; 28.2%]
	10–20%	6	18.2% [7.0%; 35.5%]
	≥ 20%	16	48.5% [30.8%; 66.5%]

*CI*—confidence interval, *N*—number

Visual field mean deviation (MD) changed from a mean − 3.8 ± 3.59 dB at baseline to − 3.3 ± 3.72 dB at 3 months, which was not statistically significant (*p* = 0.17). Pattern standard deviation (PSD) changed from a mean 4.4 ± 3.34 dB to 4.4 ± 3.48 dB at 3 months, which was also not statistically significant (*p* = 0.76). Best-corrected distance visual acuity (BCDVA) changed not statistically significantly from 0.1 ± 0.17 logMAR by 0.0 ± 0.11 logMAR at 3 months (*p* = 0.63).

Five treatment naïve eyes (15%) and 29 pre-treated eyes (85%) were included in the study. The two subgroups had neither a statistically significantly different IOP at baseline (*p* = 0.12), nor at 1 and 3 months (*p* = 0.35 and *p* = 0.70, respectively).

### Adverse events

Three patients (8.8%) had a 1 h-postoperative IOP-spike ≥ 5 mmHg from baseline IOP, which resolved within 1 day. Six patients (17.6%) experienced eye pain or discomfort, which was mainly due to the contact lens. Furthermore, there was 1 case (2.9%) of conjunctival hyperaemia and one case (2.9%) of photophobia. All of these events were transient and could be resolved without sequelae. No serious adverse events, such as uveitis, corneal edema, choroidal effusion, peripheral anterior synechiae or cystoid macular edema occurred among the study population.

## Discussion

In this prospective, interventional multicenter clinical investigation, medically treated (*n* = 29) as well as treatment naïve eyes (*n* = 5) of patients with POAG underwent SLT with the VISULAS^®^ green laser. At 3 months, 78.8% showed an IOP reduction using the same (pre-treated group) or no topical IOP-lowering (treatment naïve group) medication as before SLT. An IOP reduction of ≥ 20% from baseline was seen in 48.5% of the cases. Adverse events were mild, transient and were resolved without sequelae. Transient IOP-spikes, eye pain or discomforts are frequently reported side effects of SLT with an incidence comparable to the present clinical investigation.

The efficacy outcome in further lowering IOP in treated POAG eyes in the current study is comparable with other reported SLT studies [15]. Success of SLT treatment is commonly defined as the proportion of eyes which achieve a reduction in IOP of  ≥ 20% from baseline without an increase in hypotensive glaucoma medication and/or repeat glaucoma laser or surgical procedure [16]. Treatment success according to this definition was achieved in 48.5% of eyes 3 months post-SLT, although baseline IOP was rather low. This is in accordance with other studies using 360° SLT in POAG eyes adjunctive to ocular hypotensive medication. In a systematic review Wong et al. [17] found a mean relative IOP reduction of − 14.7% from a baseline IOP of 21.3 ± 4.7 mmHg with 40.3% showing a ≥ 20% IOP reduction 12 months post-SLT. In a retrospective study Chadwick et al. [18] found a mean relative IOP reduction of −16.7% from a baseline IOP of 20.9 ± 5.1 mmHg 3 months post-SLT, which is comparable to the current study. In a prospective study, Kuley et al. [19] determined an IOP reduction from 19.6 ± 5.2 mmHg at baseline to 16.6 ± 5.3 at 3 months with 22,8% showing a ≥ 20% IOP reduction at 3 months. This is worse in comparison with the current study and the study by Wong et al. [17, 19]. Mean baseline IOP was only 19.6 ± 5.2 mmHg in the Kuley study, however. Up to now, the only consistently reported variable that predicts a better IOP-lowering effect after SLT is a higher IOP at baseline [10, 16, 20]. This trend was also found in the present study cohort.

Nevertheless, SLT is not effective in all treated eyes. In accordance with other studies [10, 15], there was a segment of non-responders seen in our cohort: seven (21.2%) patients had no IOP reduction 3 months post-SLT. The reason as to why a considerable proportion of eyes do not adequately respond to SLT is still inconclusive. There is increasing evidence that POAG probably affects not only the trabecular meshwork (TM) but also post-TM structures like Schlemm´s canal and the collector channels [21]. A genetic variability also plays a role [21]. A TM-targeted therapy, such as SLT, is not effective if outflow resistance is rather due to Schlemm´s canal or collector channel pathology [22].

Observed procedure-related ocular adverse events included mild and self-limiting ocular discomfort or pain and postoperative IOP-spikes. Most cases of eye pain and discomfort occurred immediately after the laser procedure and can be attributed to the use of the contact lens. Ocular discomfort or pain as well as in most cases, mild anterior chamber inflammation is commonly reported after SLT [15, 16, 20, 23]. A mild inflammatory response is usually transient and resolves itself within a few days [23]. Nevertheless, the regular use of post-SLT anti-inflammatory treatment is not recommended [24]. The occurrence of transient IOP-spikes is also a commonly reported side effect of SLT and was seen in 8.8% of the eyes in the current study, which is within the range reported by other SLT studies. In a systematic review by Wong et al. [15] the incidence of IOP-spikes varied from 0 to 28.8%. Similarly, Latina et al., reported that transient IOP-spikes of 5 mmHg or more occurred in 24% of cases [25]. All of these spikes had already disappeared after one day. No serious side effects or adverse events occurred in the current study. In particular, there were no cases such as severe uveitis, corneal edema with stromal haze, choroidal effusion, hyphema or peripheral anterior synechiae, retinal complications such as cystoid macular edema or macular burns. Although rare, all of these complications have been reported following SLT [15, 23].

The present study has several limitations. Most importantly, the follow-up period of 3 months was relatively short. Therefore, long-term treatment effects and long-term complications cannot be evaluated. An additional observational phase which is intended to collect further clinical data from study participants at 6, 9 and 12 months is ongoing. Furthermore, it was designed as a single-arm study without a control group. Though it meets the calculated sample size, 34 eyes is a rather low number of patients. The study population consisted only of White/European patients with a majority of them having a mild to moderate trabecular meshwork pigmentation. Finally, it enrolled only patients with POAG. The conclusions drawn may not apply to other ethnicities or glaucoma entities. Further studies with a higher number of patients, different ethnicities and longer follow-up are recommended.

An important strength of the present study is that measurements of IOP were taken within a narrow time frame of the same 2 h of the day for each patient at each study visit using a Goldmann tonometer. Three consecutive measurements were performed in a masked, 2-person method and the median was taken.

In conclusion, SLT with the VISULAS^®^ green laser demonstrated clinically significant efficacy in terms of lowering IOP in eyes with POAG with about half of the treated eyes showing a 20% additional IOP reduction. Success rates are comparable to success rates described in literature using other conventional SLT lasers. SLT with VISULAS^®^ green showed a good safety profile in line with other reports on SLT safety.
